# Evidence of Some Natural Products with Antigenotoxic Effects. Part 1: Fruits and Polysaccharides

**DOI:** 10.3390/nu9020102

**Published:** 2017-02-02

**Authors:** Jeannett Alejandra Izquierdo-Vega, José Antonio Morales-González, Manuel Sánchez-Gutiérrez, Gabriel Betanzos-Cabrera, Sara M. Sosa-Delgado, María Teresa Sumaya-Martínez, Ángel Morales-González, Rogelio Paniagua-Pérez, Eduardo Madrigal-Bujaidar, Eduardo Madrigal-Santillán

**Affiliations:** 1 Instituto de Ciencias de la Salud, Universidad Autónoma del Estado de Hidalgo, Ex-Hacienda de la Concepción, Tilcuautla, Pachuca de Soto 42080, Hidalgo, México; jizquierdovega@gmail.com (J.A.I.-V.); spmtz68@yahoo.com.mx (M.S.-G.); gbetanzo@uaeh.edu.mx (G.B.-C.); 2 Escuela Superior de Medicina, Instituto Politécnico Nacional, Unidad Casco de Santo Tomas, Plan de San Luis y Díaz Mirón s/n, México D.F. 11340, México; jmorales101@yahoo.com.mx (J.A.M.-G.); sara.odet26@gmail.com (S.M.S.-D.); 3 Secretaría de Investigación y Estudios de Posgrado, Universidad Autónoma de Nayarit, Ciudad de la Cultura Amado Nervo. Boulevard Tepic-Xalisco s/n, Tepic 28000, Nayarit, México; teresumaya@hotmail.com; 4 Escuela Superior de Cómputo, Instituto Politécnico Nacional, Unidad A. López Mateos, Av. Juan de Dios Bátiz. Col., Lindavista, México D.F. 07738, Mexico; anmorales@ipn.mx; 5 Laboratorio de Bioquímica Muscular, Instituto Nacional de Rehabilitación, Av. México-Xochimilco. Col., Arenal de Guadalupe, México D.F. 14389, México; rogelpp@yahoo.com; 6 Escuela Nacional de Ciencias Biológicas, Instituto Politécnico Nacional, Ciudad de México, Unidad A. López-Mateos, Av. Wilfrido Massieu s/n, Lindavista, México D.F. 07738, México; eduardo.madrigal@lycos.com

**Keywords:** antigenotoxic, fruits, polysaccharides, chromosomal aberrations, cancer, micronucleus, comet assay

## Abstract

Cancer is one of the leading causes of deaths worldwide. The agents capable of causing damage to genetic material are known as genotoxins and, according to their mode of action, are classified into mutagens, carcinogens or teratogens. Genotoxins are involved in the pathogenesis of several chronic degenerative diseases including hepatic, neurodegenerative and cardiovascular disorders, diabetes, arthritis, cancer, chronic inflammation and ageing. In recent decades, researchers have found novel bioactive phytocompounds able to counteract the effects of physical and chemical mutagens. Several studies have shown potential antigenotoxicity in a variety of fruits. In this review (Part 1), we present an overview of research conducted on some fruits (grapefruit, cranberries, pomegranate, guava, pineapple, and mango) which are frequently consumed by humans, as well as the analysis of some phytochemicals extracted from fruits and yeasts which have demonstrated antigenotoxic capacity in various tests, including the Ames assay, sister chromatid exchange, chromosomal aberrations, micronucleus and comet assay.

## 1. Introduction

Genotoxicity is the ability of different agents to produce damage to genetic material. However, the damage induced in the genetic material includes not only DNA, but also all those cellular components related to the functionality and behavior of chromosomes within the cell. An example of this are the proteins involved in the repair, condensation and decondensation of DNA in the chromosomes, or other structures as the mitotic spindle, responsible for distribution of the chromosomes during cell division [1,2,3]. The agents capable of causing genetic toxicity are described as genotoxic or called genotoxins; and according to their origin, they are classified into three categories: physical, chemical and biological. The first category includes the ionizing and electromagnetic radiation, temperature and ultraviolet light. The second group consists of a wide range of compounds with multiple effects, highlighting the heavy metals, pesticides, aromatic hydrocarbons, alkylating agents, acridine, acrylamide, aliphatic epoxides, organic solvents, asbestos particles, food additives and xenobiotics resulting from certain “lifestyles” such as smoking or drinking (alcoholism). The last category considers some parasites, bacteria, plants, viruses and fungi (specifically those that synthesize secondary metabolites such as mycotoxins) [3,4,5].

**Figure 1:**
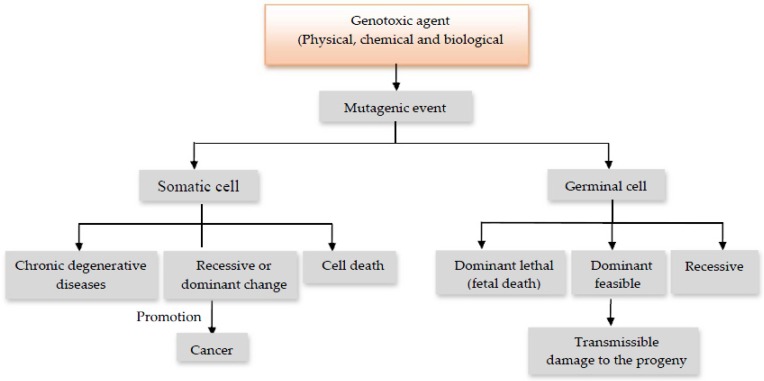
Effects produced by genotoxic agents [6].

At the same time, genotoxic agents may also be classified according to their effects or mode of action into mutagens, carcinogens or teratogens, resulting in three types of processes: mutagenesis, carcinogenesis and teratogenicity. Mutagenesis considers, basically, two types of genetic alterations. Alterations (mutations) that may occur at the level of a minimum unit of information (gene) or higher-level units, such as structural groups (chromosomes), to what is called micromutation or macromutation, respectively [2,3]. In the case of macromutations, the clastogenic agents are defined as those capable of inducing chromosome breaks and the aneunogen agents, those who produce the loss of whole chromosomes or chromosome sets. Mutations may occur on somatic and/or germ cells, being in the latter case inheritable if they are transmitted to the progeny. There is increasing evidence that mutation in somatic cells are not only involved in the carcinogenesis but can also cause genetic disorders such as arteriosclerosis, heart diseases and several other chronic degenerative diseases (Figure 1) [1,4,6].

Carcinogenesis is a process that involves changes such as irreversible cell transformation through a series of stages (initiation, promotion and progression). It has been observed that 90%–95% of carcinoma cases are associated with chemical agents, 5%–10% with physical agents and between 2%–5% with biological agents. Moreover, teratogenesis involves the induced damage in the organism development; that is to say, at any time during the gestation period [2,3,4]. It is important to remember that the ability to induce damage of these genotoxic agents is influenced by the dose, time or route of exposure, together with the genetic constitution of the individual that can define susceptibility.

Since the genotoxic agents are involved in the initiation and promotion of several human diseases, the significance of novel bioactive phytocompounds in counteracting these mutagenic and carcinogenic effects is now gaining credence. Such chemicals that reduce the mutagenicity of physical and chemical mutagens are referred to as antimutagens. However, taking into account that all mutagens are genotoxic, but not all genotoxic substances are mutagenic, the compounds that reduce the DNA damage caused by genotoxic agents are also called antigenotoxic agents [1,7,8].

Numerous studies have been carried out in last decades in order to identify compounds that might protect humans against DNA damage and its consequences. There are continual efforts all over the world to explore the rich biodiversity of edible (fruits, vegetables) as well as medicinal plants and other edible non-toxic plants in pursuit of the most effective phyto-antimutagens. These bioactive compounds belong to a variety of different chemical groups such as phenolics, pigments, allyl sulfides, glucosinolates, tannins, anthocyans, flavonoids, phytosterols, protease inhibitors and phytoestrogens [7,8]. Many of these substances, apart from their antimutagenic and anticarcinogenic properties, have shown other beneficial effects for health, such as immunomodulator, hepatoprotective, antihyperglycemic, antihyperlipidemic, cardioprotective, anti-inflammatory and antirheumatic actions owing to their excellent antioxidant and detoxifying properties [1].

In general, the antimutagens have been classified as desmutagens and bio-antimutagens; the first group considers substances that promote the elimination of the genotoxic agent from the organism as well as substances that inactivate the mutagens partially or fully by enzymatic or chemical interaction before the mutagen attacks the genes (These must be considered only as apparent antimutagens). On the other hand, the bio-antimutagens (also known as true antimutagens) can suppress the process of mutation after genes are damaged by mutagens. They act on the repair and replication processes of the mutagen-damaged DNA resulting in a decline in mutation frequency [7,8].

The mechanisms of action of the antigenotoxic agents are complex and can be categorized according to the site of action or by the specific type of action. An obvious approach is to avoid exposure to recognized risk factors. However, complementary strategies are to render the organism more resistant to mutagens/carcinogens and/or to inhibit progression of the chronic disease by administering chemopreventive agents. In a primary prevention setting, addressed to apparently healthy individuals, it is possible to inhibit mutation and cancer initiation by triggering protective mechanisms either in the extracellular and intracellular environment, e.g., modifying transmembrane transport, modulating metabolism, blocking reactive species, inhibiting cell replication, maintaining DNA structure, modulating DNA metabolism and repair, and controlling gene expression. Tumor promotion can be counteracted favoring antioxidant and anti-inflammatory activity, inhibiting proteases and cell proliferation, inducing cell differentiation, modulating apoptosis and signal transduction pathways. In a secondary prevention setting, when a premalignant lesion has been detected, it is possible to inhibit tumor progression via the same mechanisms or through affecting the hormonal status, the immune system and inhibiting tumor angiogenesis. Finally, in the tertiary prevention (strategy considered outside to the classical definition of chemoprevention) addressed to cancer patients after therapy, explores similar mechanisms, highlighting the possibility to affect cell-adhesion molecules and to activate antimetastasis genes [7,9]. The main chemopreventive mechanisms along with some examples of dietary antimutagens are shown in Table 1.

Genetics toxicology is a multidisciplinary science that studies the interaction of physical, chemical and biological agents with the genetic material, the response mechanisms to the damage and their impact on the organisms. Due to its wide application in environmental and human monitoring, it has also been used to evaluate the antigenotoxic effects of the plants, vegetables, fruits and substances of recent formulation. There are different assays, in vitro and in vivo (Table 2), to determine the genoprotector capacity of the compounds; therefore, it would be complicated to describe in detail each one. Each test has its advantages and disadvantages, but overall, the use of sensitive assessment methods that are rapid, simple and able to evaluate the genotoxic and antigenotoxic effect on somatic and germ cells as well as proliferating and non-proliferating cells have been considered [2,10,11]. Among the tests that most excelled in last decades, we can mention the bacterial mutation assay (Ames test), sister chromatid exchange, evaluation of chromosomal aberrations, micronucleus assay; and more recently, the comet assay or single cell electrophoresis [12,13,14,15,16].

There are countless studies in this field, though all of them would be impossible to mention; however, we can refer to some in order to illustrate the usefulness of the antigenotoxic tools used in the population. This present review aims to gather a good deal of data based on works conducted in some fruits frequently consumed by humans that have demonstrated antigenotoxic capacity, as well as analysis of some phytochemicals extracted from fruits, and yeasts that have been evaluated in five of the different models used in genetic toxicology (salmonella mutagenicity test, sister chromatid exchange (SCE), chromosomal aberrations, micronucleus and comet assay). With these goals in mind, the authors of this paper attempt to provide information and bibliographic support to researchers who are exploring compounds with this potential and to encourage the development of new investigations in this area of study.

## 2. Antigenotoxic Fruits

### 2.1. Pomegranate (Punica granatum L.)

**Overview:** The pomegranate (*Punica granatum* L.) is native to the Himalayas in northern India to Iran but has been cultivated and naturalized since ancient times over the entire Mediterranean region. This fruit was introduced to the American continent by Spanish missionaries during the conquest, who cultivated it in warm and arid areas of the United States and Mexico. Specifically in Mexico, the crop is produced commercially in the states of Hidalgo and Guanajuato [17].

The pomegranate (PG), an ancient, mystical, and highly distinctive tree, is a predominant member of two species comprising the *Punicaceae* family. The tree typically grows up to 12–16 feet in height and has many spiny branches. The PG can be divided into several anatomical compartments including seed, fruits, juice, peel, leaves, flowers, bark, and root. The edible fruit is a berry which is about 5–12 cm in diameter with a rounded hexagonal shape, with a thick reddish skin and around 600 seeds, each surrounded by a water-laden pulp (aril) ranging in color from white to deep red or purple. The aril is the edible part of the fruit [18].

In diverse parts of the world, including its countries of origin, the fruit is consumed fresh; but in the last years, the production of packaged juice has gained relevance. Other important PG products are marmalades, ice cream, creams and gels [19].

Its therapeutic properties are extensive and have been found in the bark, leaves, flowers and fruit (mainly, juice, seeds and peel). PG has been used as a traditional remedy against acidosis, dysentery, microbial infections, diarrhea, helminth infection, hemorrhage and respiratory pathologies. It has also been used in the treatment and prevention of cancer, cardiovascular disease, diabetes, dental conditions, erectile dysfunction, arthritis, obesity, and protection from ultraviolet (UV) radiation. Over the past decade, significant progress has been made in establishing the pharmacological mechanisms of PG and the individual constituents responsible for them. Current research seems to indicate the most therapeutically beneficial PG constituents, which are ellagic acid ellagitannins (including punicalagins), punicic acid, flavonoids, anthocyanidins, anthocyanins, and estrogenic flavonols and flavones [20].

**Antigenotoxic evidence of the fruit and its main phytochemicals (punicalagin):** Despite the common consumption of the PG as a fresh fruit and packaged juices, there are only a few studies that analyze its protective effects against the damage produced by genotoxic substances. The main evidence that suggests its antigenotoxic potential has emerged due to its high antioxidant capacity. One of the first studies was the investigation performed by Alekperov (2002) [21], who evaluated the antimutagenic effect of the bioactive compounds of the fruits of *Punica granatum* L., *Morus alba* L., and *Cydonia oblonga* Mill., on mutations induced by genotoxicants such as X-rays, *N*-methylnitrosourea, and cyclophosphamide (CP) in bone marrow cell chromosomes of mice and rats. Their results were very brief and only concluded that the plant products showed an ability to decrease the frequency of chromosome aberrations, and that the antimutagenic properties of the complex mixtures were considerably greater than those of the separate components [21]. These results motivated Sánchez-Lamar et al. [22] to analyze the protective effect of an extract from PG against the damage induced by hydrogen peroxide in ovarian cells of Chinese hamsters cultured in vitro. In this study, it was determined that the extract showed a powerful antioxidative activity which significantly reduces the frequency of sister chromatid exchange induced by the toxic agent.

It was not until 2012 that the phytochemical compounds and the antigenotoxic potential of a *Punica granatum* leaf extract (PLE) were analyzed. On this occasion, the phytochemical analysis showed the presence of flavonoids, phenols, phytosterols, tannins, and carbohydrates. With respect to the antigenotoxic capacity, this property was assessed by means of a mouse bone marrow micronucleus test. Three test doses (400, 600 and 800 mg/kg body weight) of PLE were administered by gavage to Swiss albino mice for seven consecutive days. Subsequently, these animals were injected intraperitoneally with cyclophosphamide (40 mg/kg). The results showed that all the doses were effective in exerting significant antigenotoxic effects against CP, observing that the maximum reduction was in the mice pretreated with a dose of 800 mg/kg [23]. Later on, a group of Iranian researchers evaluated the neuroprotective property of the PG using serum/glucose deprivation (SGD) as an in vitro model for the understanding of the molecular mechanisms of neuronal damage during ischemia. The aim was to investigate the possible protective effects of different extracts of pomegranate against SGD-induced PC12 cell injury. Initially, the cells were pretreated with different concentrations of pulp hydroalcoholic extract, pulp aqueous extract and PG juice, and then deprived of serum/glucose. SGD caused a significant reduction in the cell viability after 6 and 12 h, when compared with control cells. The results indicated that the pretreatment with different extracts of PG increased the cell viability significantly. In conjunction with these data, an increase in DNA damage (measured by the comet assay) in the nuclei of cells subject to SGD was observed. In contrast, the same pretreatment showed a significant decrease in DNA damage following an ischemic insult. The researchers concluded that the cytoprotective property of different extracts under a SGD condition suggest that PG has the potential to be used as a new therapeutic strategy for neurodegenerative disorders, and might be regarded as a genoprotective agent [24].

Whereas the major ellagitannin found in the fruit peel of *Punica granatum* is the punicalagin (PC), Zahin et al. [25] evaluated the antimutagenic potential, inhibition of benzo(*a*)pyrene-induced DNA damage, and antiproliferative activity of PC. They observed that the tested doses of PC (50–500 μM) exhibited significant antimutagenicity against the toxic effect of benzo[*a*]pyrene. Also, a profound antiproliferative effect on human lung cancer cells was observed. Their data suggest that the PC could be therapeutically evaluated in a suitable animal model. Recent studies performed by our research group evaluated the antigenotoxic potential of a microencapsulated pomegranate (MEPG) against damage caused by acrylamide (AA) in the micronucleus assay. Considering that PG is a seasonal fruit and it is difficult to consume all year, a microencapsulated (conversion of natural juice in small particles of water-soluble powder) was designed to maintain its natural properties and allow their antioxidant compounds to reach the digestive tract. The MEPG was administered intragastrically for 14 days before the intraperitoneal administration of AA. During one week, we performed a blood smear that was stained and observed microscopically to quantify the number of micronucleated erythrocytes normochromic (MNNE). Our results indicated that the genotoxic effect of AA increased with the administration period, whereas the MEPG showed not to be a micronucleus inducer agent and significantly reduces the frequency of MNNE at the end of the experiment (40%) [26].

Although the pomegranate is considered a fruit with high antioxidant capacity, some experiments developed by Sánchez-Lamar et al. [22] showed contradictory results in comparison with the previously mentioned studies. This group of researchers evaluated the genotoxicity of a *P. granatum* whole fruit extract using different in vitro (Ames test, cytogenetic evaluation on CHO cells, sister chromatid exchanges and chromosome aberration) and in vivo (mouse bone marrow micronucleus and sperm-shape abnormality) assays to detect DNA damage at different expression levels. Their results showed that a hydroalcoholic extract of *P. granatum* whole fruits was genotoxic when tested both in vitro and in vivo. In this sense, interestingly, certain compounds exhibit dual nature and display both antimutagenic and mutagenic effects. Such substances or compounds are known as “Janus mutagens”, in honor of the Roman god who had one head with two faces looking in opposite directions [1].

### 2.2. Guava (Psidium guajava L.)

**Overview:** *Psidium guajava* L., known as guava, is a medicinal plant belonging to the family Myrtaceae. It is a small tree, which grows up to 20 feet in height. Leaves are opposite, oblong, three to seven inches in length, with prominent veins below. Flowers are white and about one inch in diameter. Its fruits are ovoid and pear-shaped with a thin shell that has many seeds embedded in a firm flesh [27]. 

The guava tree and its fruit are considered native to Mexico and are an important food crop available in tropical and subtropical countries. It is widely used in food and folk medicines around the world. Just as the pomegranate, the guava is traditionally consumed as fresh fruit, marmalades, sweets, and salads [27,28].

Guava is a well-known traditional medicinal plant used in various indigenous systems of medicine (Table 3). The main traditional use is as an antidiarrheal (antispasmodic) agent; but because of its broad spectrum of phytochemicals that includes minerals, enzymes, proteins, triterpenes, alkaloids, glycosides, steroids, gallocatechin, flavanoids, tannins, saponins, quercetin, vitamins, carotenoids, lectins, leucocyanidin, ellagic acid, beta-sitosterol, lutein, zeaxanthine and lycopene, its therapeutic usages have increased, highlighting its potential antioxidant, hepatoprotective, antiallergic, antimicrobial, antiplasmodial, antidiabetic, anti-inflammatory and antinociceptive benefits [27,29].

**Antigenotoxic evidence of the fruit and its main phytochemicals:** Just as PG, guava is a highly consumed fruit and it has many uses in traditional medicine attributed to different phytochemicals with antioxidant properties present in its chemical composition. Unfortunately, there is very little scientific evidence in the literature of its potential antigenotoxic ability and the majority of this evidence is related to in vitro assays. These studies began in the 1990s and the antimutagenicity of water and chloroform extracts of guava against direct-acting mutagens (such as 4-nitro-o-phenylenediamine, sodium azide, and 2-aminofluorene) in the strains of *Salmonella typhimurium* were initially evaluated, thereby determining that aqueous extracts have better antimutagenic action. In the same way, a (+)-gallocatechin isolated from an extract of guava leaves was evaluated against UV-induced mutation in *Escherichia coli* confirming again its antimutagenic capacity. The results of both studies conclude and suggest that aqueous extract and phytochemicals can be considered desmutagens [30,31]. It was in 2004 when studies resumed; the first was aimed to evaluate *Psidium guajava* L. (PSG) and *Achillea millefolium* L. infusions on chromosomal aberration formation in human lymphocyte system in vitro associating them with the alkylating agent mitomycin C (MMC) and the DNA repair inhibitor cytosine-beta-arabin-furanoside (Ara-C) [32]. The results showed that the PSG infusion did not cause a clastogenic effect compared to the negative control; nevertheless, the aberrant cell frequency after MMC and Ara-C treatment was elevated. On the contrary, the PSG infusion caused a significant reduction in aberrant cell frequency produced by both mutagens. These results suggest that the infusion can influence the clastogenic action of MMC and Ara-C on DNA break induction in vitro.

Using the same genotoxicant (MMC), nalidixic acid and hydrogen peroxide; Bartolome et al. [33] evaluated in an optical antigenotoxicity assay (called “SOS-red fluorescent protein (RFP) bioassay system”) the protective ability of aqueous extracts of *Mangifera indica*, and *Psidium guajava* L. against these three agents. The antigenotoxic potential of the extracts was shown to inhibit the genotoxicant-triggered red fluorescence produced by the mutagens. Finally, Taiwanese researchers examined the inhibitory effects of the aqueous extract from guava twigs (GTE) on mutation and oxidative damage produced by 4-nitroquinoline-N-oxide, a direct mutagen, and 2-aminoanthracene, an indirect mutagen, in *Salmonella typhimurium* TA 98 and TA 100. Their results show that GTE inhibits the mutagenicity of both mutagens and using high-performance liquid chromatography analysis found that the major phenolic constituents in GTE are gallic acid, ferulic acid, and myricetin, which may contribute to the inhibitory effect [34].

Two in vivo studies, using the comet assay, have been performed so far with the goal to evaluate the antigenotoxic potential of the guava. In the first, one, the effect of two doses (125 and 250 mg/kg) of a *Psidium guajava* L. (PSG) supplementation orally administered for four weeks against DNA damage produced by streptozotocin was analyzed. Another finding was that at the end of the study the supplementation of PSG restored the loss of body weight and significantly reduced the DNA strand breaks induced by streptozotocin [35]. The protection observed in the DNA of pancreatic cells was approximately 90% in both doses. In a second study, the radioprotective activity of hydroalcoholic leaf extracts of *Psidium guajava* against rats exposed to X-rays was analyzed. The best protective effect was obtained with a dose of 200 mg/kg (a similar dose to that used in the previous study). The animals pre-treated with the extract showed a reduction in olive tail moment and lower percentage of DNA in tail compared to the irradiated group alone [36].

### 2.3. Grapefruit (Citrus paradisi Macfad)

**Overview:** The grapefruit is an important member of the genus *Citrus* of the Rutaceae family; its scientific name is *Citrus paradisi*. The grapefruit originated on Barbados Island, but it is currently cultivated in Mexico, Spain, Morocco, Israel, Jordan, South Africa, Brazil, and Jamaica and in the Asian continent [37]. In addition, it is consumed as a seasonal fruit or as grapefruit juice to accompany other foods, it has also been used in many countries both in popular and traditional medicine as an antimicrobial, antifungal, anti-inflammatory, antioxidant, and antiviral, as well as an astringent solution, and as a hepatoprotective agent [38]. Studies conducted over past decades have suggested that the grapefruit might be active in cellular regeneration, cholesterol reduction, the detoxifying process, and the maintenance of heart health, as well as rheumatoid arthritis, the control of body weight, and cancer prevention [38].

Grapefruit juice is an excellent source of many phytochemicals and nutrients that contribute to a healthy diet. It contains significant levels of vitamin C, folic acid, phenolic acid, potassium, calcium, iron, limonoids, terpenes, monoterpenes, and d-glucaric acid. The red and pink varieties also contain beta-carotene and lycopene, which are antioxidants that the body can convert into vitamin A [37]. However, the flavonoid that has the greatest concentration is naringin, which is metabolized into naringenin by the human body [39,40,41].

**Antigenotoxic evidence of the fruit and its main phytochemicals (naringin and naringenin):** Several studies have shown that grapefruit is a good plant-based source of vitamins and three significant groups of phytochemicals (limonoids, lycopene and flavonoids). For this reason, there is a great scientific interest in studying their biological effects, especially its DNA protective capacity. 

Diverse studies have been developed since 2001, all of which have shown that the fruit, juice and two of its phytochemicals are good candidates to be considered antigenotoxic agents. The results suggest that the grapefruit may reduce the frequency of chromosomal aberrations, sister chromatid exchange (SCE) and micronuclei, as well as single or double strand breaks of DNA produced by 2-amino-1-methyl-6-phenylimidazo (4,5-*b*) pyridine, aflatoxin B_1_ (AFB_1_), daunorubicin, ifosfamide, benzo(*a*)pyrene, hydrogen peroxide (H_2_O_2_), amiodarone, and X-rays. In the case of the naringin, this compound has shown its genoprotector capacity against the ifosfamide, H_2_O_2_, cytosine-beta-arabin-furanoside (Ara-C), bleomycin, and cadmium (Table 4) [42,43,44,45,46,47,48,49,50,51,52,53,54,55].

It is important to remember that the mechanisms of action of the chemopreventers are complex and can be categorized according to the site of action or by the specific type of action. Likewise, many antimutagenic compounds can display multiple mechanisms (Table 1). In this sense, the grapefruit and its main phytochemicals (naringin and naringenin) not only have shown their antioxidant capacity, but there is also evidence of their potential to influence the biotransformation and absorption of some mutagens.

It has been observed that grapefruit juice intake, as well as naringin and naringenin, significantly suppress AFB_1_-induced liver genotoxicity through inhibiting the activity of the cytochrome P450 in rats (specifically, by reducing of CYP3A4 expression). Considering that CYP3A4 is an important enzyme in the body and mainly found in the liver and in the intestine, it has been observed that its inhibition at this level may amplify or weaken the action of some drugs, mutagens and carcinogens [43,56,57].

On the other hand, two studies have been considered relevant to demonstrate the ability of grapefruit and its phytochemicals to reduce the absorption of certain toxic compounds. In the first one, the effect of grapefruit juice (GFJ) on drug transport by MDR1 P-glycoprotein (P-gp) and multidrug resistance protein 2 (MRP2), which are efflux transporters expressed in human small intestine, was analyzed. The transcellular transport and uptake of vinblastine (VBL) and saquinavir in a human colon carcinoma cell line (Caco-2) and in porcine kidney epithelial cell lines transfected with human MDR1 cDNA, LLC-GA5-COL150, and LLC-MRP2 were also examined. Their results revealed that GFJ interact with not only P-gp but also MRP2, both of which are expressed at apical membranes and limit the apical-to-basal transport of VBL and saquinavir in Caco-2 cells [58]. The second study, developed by Romiti et al. [59], evaluated the effect of GFJ, kaempferol and naringenin on the expression and activity of Pgp in the human immortalized tubular cell line HK-2 using Western blot analysis and RT-PCR. At the end of the experiment, they confirmed a downregulation of Pgp as well as inhibition of its function by GFJ or its related components in tubular cells. In conclusion, both studies confirmed that the multidrug transporter MDR-1 P-glycoprotein is an important mechanism of absorption whose modulation depends on the chemical interaction between drugs and many commonly ingested substances, including grapefruit juice.

### 2.4. Pineapple (Ananas comosus L.)

**Overview:** The pineapple (*Ananas comosus* L.), traditionally known as the “king of fruit” because of its crown of leaves, is a perennial monocot belonging to the family Bromeliaceae. It is a tropical plant native to South America and the third most important tropical fruit crop after banana and mango [60,61]. The pineapple is the only available edible bromeliad today. It is a multiple fruit, actually composed of several individual flowerets that grow together to form the entire fruit. Due to its attractive sweet flavor, the pineapple is widely consumed as a fresh and canned fruit, as well as in processed juices and as an ingredient in exotic foods [62]. This fruit contains micronutrients, such as vitamin C, manganese, thiamin, riboflavin, pyridoxine, copper, and dietary fiber. It also contains different phytochemicals, i.e., ascoumaric acid, ferulic acid, chlorogenic acid, ellagic acid, and bromelain [61,62]. Some studies have shown that the regular intake of pineapple may increase the consumption of these phytochemicals and micronutrients and potentially influence some immunological markers and help improve a child’s physical health [61,63]. This fruit has been widely used as a therapeutic plant in several resident cultures and its therapeutic qualities are mainly related to the bromelain enzyme, which is an elementary extract from pineapple that contains, along with other compounds, various proteinases. Bromelain has shown to exhibit various fibrinolytic, antiedematous, antithrombotic, and anti-inflammatory activities both in vitro and in vivo [63,64]. Ever since bromelain was known chemically, it has been used as a phytomedical agent. To confirm its ubiquitous nature, various clinical studies have been conducted, and the results have shown that bromelain may help the treatment of several diseases such as cardiovascular disease, osteoarthritis, diarrhea, and cancer. It has been widely used in debridement of burns, coagulation, and immunogenicity [63,64].

**Antigenotoxic evidence of the fruit and its main phytochemical (bromelain):** This is another fruit that has garnered little scientific interest to explore its DNA protective capacity. The genoprotective effect of *Ananas comosus* L. was first examined by Ikken et al. (1999) [65], who evaluated the antimutagenic ability of fruit and vegetable ethanolic extracts against N-nitrosamines (including to *N*-nitroso dimethylamine (NDMA), *N*-nitroso pyrrolidine (NPYR), *N*-nitroso dibutylamine (NDBA), and *N*-nitroso piperidine (NPIP)) in the Ames test. They found that licorice and kiwi ethanolic extracts showed the best inhibitory effect against mutagenicity of all *N*-nitrosamines tested. Regarding the pineapple, a moderate protective effect was observed at the range 50–2000 µg/plate against NDMA, NPYR, NDBA and NPIP. Their results suggest that the observed differences in the antimutagenic activity of the fruits and vegetables tested could be attributed to the different antioxidant compounds present in its nutritional composition, thereby indicating that these ethanolic extracts could be useful to humans for their chemopreventive purposes [65].

Subsequently, some studies conducted by Platt et al. (2010) [46] again suggested the pineapple to have a weak protective effect in comparison to other fruits. In this case, the researchers analyzed through the comet assay the effect of three tea herbs, two types of wine, 11 different vegetables and the juice of 15 fruits on the genotoxicity induced by heterocyclic aromatic amines (HAAs). The amines evaluated were 2-amino-3-methylimidazo (4,5-*f*) quinoline (IQ) and 2-amino-1-methyl-6-phenylimidazo (4,5-*b*) pyridine (PhIP). To mimic the human metabolic activation of these HAAs, genetically engineered V79 Chinese hamster fibroblasts were employed that express human cytochrome P450-dependent monooxygenase (hCYP) 1A2 combining it with human *N*(*O*)-acetyltransferase (hNAT) 2*4 for the effect of IQ activation and (i.e., V79-hCYP1A2-hNAT2*4). For the effect of PhIP activation, it was combined with human sulfotransferase (hSULT)1A1*1 (i.e., V79-hCYP1A2-hSULT1A1*1). The analysis of the different juices showed that sweet cherry juice was the most effective against the genotoxicity of IQ, followed by juices from kiwi fruit, plum and blueberry. The juices from watermelon, blackberry, strawberry, black currant, and Red Delicious apple showed moderate suppression, whereas sour cherry, grapefruit, red currant, and pineapple juices showed only a weak activity. All fruits showed a lower inhibitory effect against genotoxicity induced by PhIP. The rest of the data indicated that black, green and rooibos tea leaves moderately reduced the genotoxicity of IQ, whereas red and white wines were less active [46]. The results of these studies are not fully conclusive on whether or not the pineapple can be used as a therapeutic plant, especially for improving the physical health of children.

The effect of pineapple peel powder addition on the viability and activity of *Lactobacillus acidophilus*, *L. casei*, and *L. paracasei ssp* in yoghurts stored at 4 °C for 28 days has recently been evaluated. The data showed significant differences between natural yogurts and others supplemented with or without pineapple peel powder. The probiotic counts and degree of proteolysis in supplemented yogurts were higher during storage. In addition, it was observed that the raw water-soluble peptide extract of the probiotic yoghurt with peel contains stronger antimutagenic and antioxidant activities (evaluated by scavenging capacity of 1,1-diphenyl-2-picrylhydrazyl; 2,2′-azino-bis(3-ethyl benzothiazoline-6-sulfonic acid), and hydroxyl radicals) compared to natural yogurts. These data open new expectations to evaluate the pineapple (as fruit or extract) against other mutagens [66].

With regard to bromelain, there is only one study that suggests its antigenotoxic potential. In this research, the bromelain ability to reduce genotoxicity of advanced glycation end products (AGEs) in mammalian cells by comet assay was evaluated. The incubation of pig kidney LLC-PK1-cells with AGEs-BSA (AGEs bovine serum albumin), carboxymethyllysine-BSA as well as methylglyoxal-BSA resulted in a significant increase in DNA damage. On the contrary, the pretreatment of the cells with the bromelain protease abolished the formation AGEs-BSA-induced comets [67].

### 2.5. Mango (Mangifera indica L.)

**Overview:** *Mangifera indica* L. (MI), also known as mango, is a juicy stone fruit belonging to the family Anacardiaceae [68,69]. It is one of the most popular fruits in the world, occupying the second position as a tropical crop, behind only bananas in terms of production and acreage used. Mango is widely distributed in the world’s tropical and subtropical regions, and it is believed to be native to India, where it was grown for more than 4000 years. More than 1000 cultivars currently exist around the world [68,70]. About 30% of world production is processed into products such as puree, nectar, slices in syrup, chutney, pickles and canned slices; the rest is consumed as fresh fruit. It has been well documented that mango fruit provides energy, dietary fiber, carbohydrates, proteins, and fats. It is an important source of micronutrients, vitamins and phytochemicals, highlighting the mangiferine, a xanthone glycoside with antioxidant capacity that has been proven to be immunomodulatory, cardiotonic, hypotensive, anti-degenerative, and anti-diabetic. In general, different parts of MI (leaves, flowers, bark, fruit, pulp, peel and seeds) have traditionally been used as a dentrifrice, antiseptic, astringent, diaphoretic, laxative and diuretic agent in addition to treating diarrhea, dysentery, anemia, asthma, bronchitis, cough, hypertension, insomnia, rheumatism, and toothache [70,71].

**Antigenotoxic evidence of the fruit and its main phytochemical (Mangiferine):** Despite being one of the most popular fruits in the world, there are only a few studies evaluating the genoprotector effect of the extract taken from the plant, pulp or fruit, compared with those that analyze the main phytochemical, the mangiferine (MGN). In relation to the mango extracts, in 2006, the SOS-red fluorescent protein (RFP) bioassay system demonstrated the antigenotoxic activity of different plant extracts, including MI. In this technique, *E. coli* RS4U was exposed to mitomycin C and H_2_O_2_, as well as to the combination of these mutagens plus *Mangifera indica* extract. The results concluded that the extract inhibited the red fluorescence that was directly proportional to the concentration of the inducer [33].

Later on, the protective effect of a mango pulp extract (MPE) against the clastogenicity produced by the benzo[*a*]pyrene (B(*a*)P) in mouse bone marrow cells was evaluated on Swiss albino mice. A significant decrease in B(*a*)P-induced clastogenicity was evidenced in the mice treated with MPE. In conclusion, the MPE can increase the mitotic index and reduce the incidence of micronuclei [72].

Finally, Cuban researchers developed two studies to evaluate the antimutagenic property of *Mangifera indica* L. stem bark extract (MSBE) against different mutagens or carcinogens. In the first study, the MSBE was tested against ten chemical mutagens with the Ames test in the absence or presence of metabolic fraction (S9). The chemicals tested included: cyclophosphamide (CP), mitomycin C (MMC), bleomycin, cisplatin, dimethylnitrosamine (DMNA), B[*a*]P, 2-acetylaminofluorene (2-AAF), sodium azide, 1-nitropyrene (1-NP) and picrolonic acid [73]. In the second study, the same extract was tested against radiation-induced DNA damage in human lymphocytes and lymphoblastoid cells evaluated with the comet assay [74]. In summary, the results indicate that: (a) MSBE (concentrations between 50 and 500 μg/plate) reduced significantly the mutagenicity mediated by all the chemicals tested with the exception of sodium azide; and (b) MSBE (25 and 50 µg/mL) reduces the gamma radiation-induced DNA damage in a concentration-dependent manner in both cell types tested.

As mentioned, the largest numbers of studies have been with the MGN. The research of this phytochemical began in 2005, and has mainly been analyzed with the comet and micronucleus assays. The scientific evidence about its antigenotoxic potential includes different evaluations in vitro and in vivo (some clinical studies in humans) against exposure to several mutagens and/or carcinogens (Table 5). The agents tested were chemical and physical, including: 2-AAF, B(*a*)P, H_2_O_2_, bleomycin, AFB_1_, sodium azide, cisplatin, MMC, DMNA, CP, cadmium chloride (CdCl_2_), methylmercury (MeHg), mercuric chloride (HgCl_2_), etoposide, and gamma radiation (oses of 1 to 5 Gy). The results conclude that MGN is not a cytotoxic or genotoxic compound; in contrast, it reduces the frequency of micronuclei and avoids single strand breaks and/or alkali-labile sites. It has antiapoptotic and radioprotective potentials. In general, the genoprotective effect may be associated with its antioxidant property and their ability to inhibit CYP enzymes (such as CYP1A1).

### 2.6. Blueberries/Cranberries (Vaccinium spp.)

**Overview:** Among the small, soft-fleshed, colorful fruits, berries make up the largest proportion consumed in the diets. These fruits are not only ingested in their fresh and/or frozen forms, but they also appear in processed products, such as canned fruits, yogurts, beverages, jams, and jellies. Thus, in recent years, the consumption of berry extracts has increased as an ingredient in functional foods and dietary supplements, which might or might not be combined with other colored fruits, plants, and herbal extracts. The berry fruits that are habitually consumed in North America include blackberries (*Rubus* spp.), black raspberries (*Rubus occidentalis*), red raspberries (*Rubus idaeus*), strawberries (*Fragaria X ananassa*), blueberries (*Vaccinium corymbosum*), and cranberries (*Vaccinium macrocarpon*) [83].

At the 2007 International Berry Health Benefits Symposium, diverse research from Asia, Europe, New Zealand, Mexico, and North and South America were presented that demonstrated the potential benefits that could be obtained by consuming these fruits. In general, it was concluded that these benefits could be observed in cardiovascular diseases, neurodegenerative diseases, and other diseases associated with aging, obesity, and some human cancers (mainly esophageal and gastrointestinal) [84]. It was determined that the agents responsible for these biological properties were diverse phenolic-type phytochemicals, among which the following were highlighted (1) flavonoids (anthocyanines, flavonols, and flavanols); (2) tannins (condensed tannins (proanthocyanidins) and hydrolyzable tannins (ellagitannins and gallotannins)); (3) stilbenoids; and (4) phenolic acids [83]. Of all of these compounds, those that have been most studied are the anthocyanins (pigments are responsible for the berries’ attractive colors), which have demonstrated antioxidant, anticarcinogenic, and anti-inflammatory biological activity [83,85].

**Antigenotoxic evidence for the berry fruits and its main phytochemicals (anthocyanins, anthocyanidins, and proanthocyanidins):** After the mango, berry fruits are the most popular fruits for study of antimutagenic and antigenotoxic properties. In the last decade, evidence of their ability to protect the DNA has increased. Several studies have been developed in vitro and in vivo aimed to assess this capacity against different mutagenic and/or carcinogenic agents. Due to the information being very extensive, the most relevant studies will be analyzed, focusing on the comet assay and micronucleus data. The studies on cell cultures and animals are mentioned first, followed by investigations developed with human samples (clinical studies), and finally, the corresponding evidence to its phytochemicals.

In relation to the first context, blueberries, blackberries and cranberries are the most commonly used species for combating the genotoxicity of different mutagens (including heterocyclic aromatic amines, alkylating agents, polycyclic aromatic hydrocarbons and ultraviolet radiation). Among the cell lines that have shown their antigenotoxic ability are Chinese hamster lung fibroblasts (V79 cells), human liver cancer (HepG2) and human keratinocyte cell line (HaCaT cells). In general, the results have shown that extracts from these fruits are not toxic in vitro and can reduce the genotoxicity of the aforementioned mutagens. Likewise, the studies suggest two important mechanisms of antigenotoxicity, such as the inhibition of enzymatic activation of xenobiotics and their ability to scavenge free radicals (Table 6).

The same table shows the results of studies developed in experimental animals. In summary, the results indicated that the extracts of blueberries and cranberries fruits were not genotoxic and cytotoxic in in vivo conditions (confirming the data observed in in vitro studies); on the contrary, they reduce the frequency of micronuclei induced by B[*a*]P, *N*-methyl-*N*′-nitro-*N*-nitrosoguanidine (MNNG) and 7,12-dimethylbenz(*a*)anthracene (DMBA). This aspect is consistent with the reduction of damage caused by H_2_O_2_ in rats fed wild blueberry and evaluated with the comet assay.

The latest evidence together with the results observed after administering a lyophilized extract of *Vaccinium ashei* berries for 30 days to the DNA damage on the hippocampus and cerebral cortex and on cognitive performance of mice, confirm the beneficial and protective property of berry fruits.

For that reason, there are some clinical studies that have evaluated the same property of these fruits (mainly consumed as juice) against some toxic compounds to which the human population is exposed. In this sense, Wilms et al. [91] evaluated the impact of multiple genetic polymorphisms on effects of a four-week blueberry/apple juice intervention on ex vivo-induced lymphocytic DNA damage in 168 healthy human volunteers. His results indicated that after a four-week intervention period, plasma concentrations of quercetin and ascorbic acid were significantly increased. Likewise, a 20% protection against oxidative damage induced by H_2_O_2_ was observed. In conclusion, the analysis of 34 genetic polymorphisms showed that DNA damage is reduced faster in some individuals due to the micronutrients in a single share of blueberry/apple juice. On the other hand, Del Bó C et al. [92] analyzed the protective effect of one portion of blueberry (*Vaccinium corymbosum* L.) on markers of oxidative stress and DNA damage induced by H_2_O_2_ in blood samples from healthy male volunteers. The results confirmed that this species of fruit significantly reduced H_2_O_2_-induced DNA damage in blood mononuclear cells and that one portion of blueberries seems sufficient to improve cell antioxidant defense against DNA damage [92].

Recently, the same group of researchers compared the DNA damage in fresh versus cryopreserved peripheral blood mononuclear cells (PBMCs) obtained from subjects following a six-week intervention with wild blueberry drink or placebo drink. The results indicated that the levels of formamidopyrimidine-DNA glycosylase (FPG)-sensitive sites were significantly higher in the cryopreserved compared with the fresh cells, while H_2_O_2_-induced DNA damage was significantly lower after storage. Also, the decrease in H_2_O_2_-induced DNA damage observed in the cryopreserved cells masked the protective effect of the wild blueberry drink documented in the fresh samples. These results suggest that H_2_O_2_-induced DNA damage could be significantly modified following the long-term storage of samples obtained from individuals participating in a dietary intervention [93].

Regarding the antigenotoxic evidence of its phytochemicals, there are many studies on anthocyanines, anthocyanidins, proanthocyanidins, ellagitannins, and quercetin, but only two relevant studies are mentioned here because they are representative of the protective potential to DNA of three phytochemicals (anthocyanins, anthocyanins, proanthocyanidins) extracted from blueberries and evaluated with the comet assay under in vitro conditions. In the first study, Philpott et al. [94] compared the antioxidant activities of extracts from six anthocyanin-rich edible plants (including the blueberries) using three chemical assays (DPPH, TRAP and ORAC). His objective was based on the consideration that anthocyanins are natural antioxidants and may reduce the susceptibility and organic damage in chronic degenerative diseases. Even though the structure-activity relationships have provided guidelines on molecular structure in relation to free hydroxyl-radical scavenging, this may not cover the situation in food plants where the anthocyanins are part of a complex mixture, and may be part of complex structures, including anthocyanic vacuolar inclusions (AVIs). The extracts showed differential effects in the chemical assays, suggesting that closely related structures have different affinities to scavenge different reactive species. The integration of anthocyanins to an AVI led to more sustained radical scavenging activity as compared with the free anthocyanin. The majority of the extracts were able to provide a significant reduction in constitutive DNA damage. Among the most significant were the blueberries [94].

The second study investigated the ability of three extracts of blueberry anthocyanins and anthocyanidins (BA) to protect cellular DNA from UV-induced damage. The extracts considered were water, ethanol and methanol. All extracts restored the proliferation of UV-irradiated HepG2 cells (evaluated by MTT assay). Treatment with BA extracts decreased reactive oxygen species and reduced DNA damage by tail moment of comet assay. The aqueous extract was the most significant to protect the DNA shown by a decrease in gene, protein expression of p53, and phospho-p53 in UV-irradiated HepG2 cells [95].

## 3. Polysaccharides

### 3.1. β-Glucans, α-Mannans and Glucomannans

**Overview:** Probiotics are defined as living microorganisms that, when administered in adequate amounts, confer a health benefit on the host. According to this concept, various species of *Lactobacillus*, *Bifidobacterium*, *Enterococcus*, and *Saccharomyces*, among other microorganisms, have been used to improve human health [96,97]. Several effects of probiotics on health have been reported, including protection against infection, reduced incidence of diarrhea, anti-microbial activity, competitive exclusion of pathogens, immune tolerance, reduction in colorectal cancer biomarkers, return to pre-antibiotic baseline flora, epithelial barrier function, increased cellular immunity, increased humoral response, reduction in irritable bowel disease symptoms, and lowering of blood cholesterol levels. Oelschlaeger [98] describes three modes of action of probiotics: (i) Probiotics might be able to modulate the host’s defences including the innate as well as the acquired immune system; (ii) They can also have a direct effect on microorganisms, commensal and/or pathogenic ones; and (iii) Probiotic effects may be based on actions affecting microbial products like toxins. Such actions may result in inactivation of toxins and detoxification in the gut. Recognizing that there seems not to be one probiotic exhibiting all modes of action, and that therefore the action of probiotic depends on the metabolic properties, the kind of surface molecules expressed and components to be secreted which probiotic actions a certain probiotic strain might show [98]. The yeast *Saccharomyces cerevisiae* (Sc), in particular, has shown stimulation of the intestinal microflorae growing in mammals, pH modulation in ruminants, an improvement in reproductive parameters (fertility and fetal development) in milk cows and chicks, as well as a reduction in the amount of pathogenic microorganisms in monogastric animals [99]. The structure of the Sc cell wall is known for including various polysaccharides, such as the structurally similar compounds glucans, mannans and glucomannans, which differ only by the type of carbohydrate and the chemical bonds. The β-glucans are mainly composed of a linear central backbone of D-glucose linked in the β (1→3) position with glucose side branches (linkage β 1→6) of various sizes. In contrast to the α-mannans constituted by a chain of mannoses linearly joined through α-1-6 bonds, and with α-1-2 and 1-3 bonds in the branches. With regard to glucomannans, its chemical structure is formed of the combination of mannose and glucose in the ratio 8:5 linked by beta (1→4) glycosidic bonds [100,101,102]. In recent years, these polysaccharides have begun to acquire great economic importance due to their immunological activity; thus, they have been classified as biological response modifiers. However, this is not the sole property that they possess because different studies have shown their antiviral, antiparasitic, antifungal, antimicrobial, antioxidant, antitumor, and antimutagenic potential [100,103,104].

**Antigenotoxic evidence for the β-glucan (Glu), α-mannan (Man) and glucomannan (Glucoman):** Several studies have demonstrated the antigenotoxic ability of these polysaccharides. However, the main research has focused on the β-glucans, especially those extracted from the cell wall of yeast, algae, bacteria and some cereals (such as barley and oats). The research group of Grigorij Kogan from the Institute of Chemistry of the Slovak Republic initiated the studies in the1990s. His research was focused on the antigenotoxic evaluation (using micronucleus test in mouse bone marrow) of three derived glucans (carboxymethylglucan, sulfoethylglucan and carboxymethyl-chitin-glucan) extracted from *S. cerevisiae* and *A. niger* against two clastogenic agents, such as cyclophosphamide and potassium bichromate. The results showed that the three derived glucans decreased the frequency of micronuclei in bone marrow cells [105,106,107]. Some years later, Oliveira et al. [108,109] explored the chemopreventive activity and mechanisms of action of β-glucan from barley in two cell lines (CHO-k1 and HTC) and in somatic cells against methylmethane sulfonate (MMS) and cyclophosphamide. The results indicated that both cell lines treated with MMS showed an antimutagenic effect at high concentrations of β-glucan (10, and 20 µg/mL) as well as a significant reduction of micronuclei of peripheral blood in male mice exposed to cyclophosphamide. The evaluation of both protocols suggested that β-glucan is capable of preventing changes in DNA and its mechanism of action may be considered as having desmutagenic and bioantimutagenic activities. These results promoted the search for new protective evidence by employing the single cell gel electrophoresis assay (also known as comet assay), different investigations were conducted (in vitro and in vivo), with the purpose of assessing the efficiency of β-glucans extracted from different fungal and cereal grains to inhibit the genotoxicity induced by diverse mutagens and/or carcinogens (direct and indirect). Evidence is concise, generally concluding that: (a) β-glucans are non-cytotoxic substances; (b) In addition to the immunopotentiating activity, they possess radioprotective activity and inhibit the genotoxicity of carcinogens with or without metabolic activation, and (c) Their antigenotoxic mechanism is related to potential free radical scavenger and their ability of adsorption to form supramolecular complexes (Table 7) [110,111,112,113,114,115,116,117,118,119,120,121,122].

Three studies have shown the antigenotoxic potential of *α-*mannan so far. The first was developed by Krizková et al. [123], who evaluated the antimutagenic activity of the yeast cell-wall mannan and mannan conjugates of *S. cerevisiae* in the unicellular flagellate *Euglena gracilis* exposed to two genotoxic agents (ofloxacin and acridine orange). All tested mannans showed a statistically significant concentration-dependent protective activity against both compounds, concluding that an important characteristic of these mannans is their high water solubility and low toxicity; these are promising properties for their practical application as antioxidants and antimutagenic agents [123].

The last two studies were developed by Madrigal et al. [101,124], who by using micronucleus, SCE and comet assays evaluated the inhibitory effect of α-mannan on the toxicity produced in mice treated with AFB_1_. The results indicated that all doses of mannan showed a significant reduction in the level of MNNE and SCE. Likewise, the analysis of comets indicated a clear prevention of DNA damage by reducing the number of grade 2 and 3 nucleoids in mouse hepatocytes [101,124].

With respect to the *glucomannans*, only one study has been undertaken, where the protective effect of this polysaccharide isolated from *Candida utilis* against cyclophosphamide-induced mutagenicity was analyzed. The frequency of micronuclei was evaluated in polychromatic erythrocytes of mouse bone marrow. The results indicated a greater decrease of micronucleated erythrocytes polychromatic (MNPE) with 200 mg/kg in comparison to 100 mg/kg, that is to say, the antimutagenic activity was dose-dependent [125].

## 4. Conclusions

The present study synthesizes the most accurate evidence of the antigenotoxic capacity of some fruits and the main polysaccharides present in the cellular wall of yeasts, algae, and cereals against different toxic compounds that cause damage to genetic materials.

In the same fashion, the investigations presented validated the use of fruits in the popular medicine and these polysaccharides as a complementary strategy to prevent cancer and other chronic degenerative diseases, some of which share common pathogenic mechanisms, such as DNA damage, oxidative stress, and chronic inflammation. It is important to remember that in order to fully confirm the antigenotoxic potential of the compounds (including mechanism or mechanisms of action), it is suggested to use several techniques considering its advantages and/or disadvantages. For example, data from comet assay should be interpreted with caution, because the strand breaks come from direct DNA damage but also from repair mechanisms (endonucleases action); therefore, a reduction in strand breakage may signify an inhibition of repair that would be the indirect genotoxicity. Likewise, the tests analyzed in this review do not completely differentiate between desmutagens and/or bio-antimutagens and it is thus important to consider more specific tests (e.g., effect on translesion synthesis). In relation to this latter mechanism, it should be mentioned that some bio-antimutagens may also act on translesion synthesis, favoring some mutagenic event (error-prone translesion synthesis) or non-mutagenic (“accurate translesion synthesis”) outcome; the reason why they can be considered “Janus mutagens” is that they exhibit a dual nature and display both antimutagenic and mutagenic effects.

## Figures and Tables

**Table 1:** Main mechanisms of antimutagenic action.

Types of Mechanisms	Examples of Dietary Antimutagens
Extracellular	
1. Inhibition of mutagen uptake	Dietary fibres, probiotics, grapefruit (naringenin).
2. Inhibition of endogenous formation *(a) Inhibition of nitrosation* *(b) Modification of the intestinal flora*	Vitamins (ascorbic acid), sulphur compounds (cysteine, glutathione). Prebiotics, probiotics.
3. Complexation and/or deactivation	Dietary fibres, chlorophyllin.
4. Favouring absorption of protective agents	Vitamin D3.
Intracellular	
5. Blocking or competition *(a) Scavenging of reactive oxygen species* *(b) Protection of DNA nucleophilic sites*	Mango (polyphenols), guava (gallocatechin) vitamins (β-carotene, α-tocopherol, ascorbic acid), pineapple, blueberries (anthocyanins). Ellagic acid, retinoids, polyamines.
6. Stimulation of trapping and detoxification in non-target cells	*N*-Acetyl cysteine.
7. Modification of transmembrane transport	Short chain fatty acids (caproate), dietary calcium.
8. Modulation of xenobiotic metabolising enzymes *(a) Inhibition of promutagen activation* *(b) Induction of detoxification pathways* *(c) Inhibition of metabolic enzymes*	Isothiocyanates, monocyclic monoterpenoids (limonene, methol), flavonoids, wheat bran. Polyphenols, indoles, diterpene esters. Grapefruit (naringin, naringenin).
9. Modulation of DNA metabolism and repair	Cinnamaldehyde, vanillin.
10. Regulation of signaling pathways	Pomegranate (polyphenols), β-glucans.
11. Enhancement of apoptosis	Retinoids, flavonoids.
12. Maintenance of genomic stability	Vitamins (folic acid, B12), minerals (selenium, zinc), polyphenols.
Table modified from Ferguson et al. (2004).	

**Table 2:** Tests used in identifying genotoxic and antigenotoxic agents.

Prokaryote and Eukaryote Models		Germinal Cell	
In vitro		In vivo	
I. Gene Mutations			
Bacteria (Ames assay, SOS chromotest) Yeast /Fungus (*S. cerevisiae assay*, *A. nidulans assay*)	Mouse spot test Somatic mutations and recombination test (SMART) DNA microarrays Serial analysis of gene expression (SAGE) Specific genes targeting		Recessive lethal Specific locus test Abnormalities of the sperm
II. Chromosome Changes			
Fibroblast culture Lymphocyte culture Mouse lymphoma assay	Micronucleus assay (MN)		Dominant lethal Heritable translocations Cytogenetic sperm Aneuploidy
III. Indicators Biological Damage			
Gene recombination Unscheduled DNA synthesis (UDS) assay	Comet assay Sister chromatid exchange (SCE) MN		Comet assay MN, SCE UDS assay

**Table 3:** Ethnomedical uses of guava in some countries [27].

Country	Traditional Uses
Amazonia	Diarrhea, dysentery, menstrual disorders, stomachache, vertigo.
Brasil	Diarrhea, anorexia, cholera, digestive problems, dysentery, gastric insufficiency, inflamed mucous membranes, skin problems, sore throat, ulcers, vaginal discharge.
Cuba	Dysentery, dispepsia.
Ghana	Diarrhea, dysentery, coughs, toothache.
Haiti	Diarrhea, dysentery, stomachache, epilepsy, itch, piles, scabies, skin sores, sore throat, wounds and as an antiseptic and astringent.
India	Anorexia, cerebral ailments, childbirth, chorea, convulsions, epilepsy, nephritis.
Malaya	Diarrhea, dermatosis, epilepsy, hysteria, menstrual disorders.
Mexico	Diarrhea, stomachache, deafnessitch, scabies, swelling, ulcer, worms, wounds.
Peru	Diarrhea, dysentery, conjunctivitis, cough, digestive problems, edema, gout, hemorrhages, gastroenteritis, gastritis, lung problems, shock, vaginal discharge, vertigo, worms.
Philippines	Sores, wounds and as an astringent.
Trinidad	Diarrhea, dysentery, bacterial infections, blood cleansing.

**Table 4:** Antigenotoxic evidence of the fruit and its main phytocchemicals.

Year	Main Objetive	Type of Study	Assay Employed	Reference
Fruit, juice or extract				
2002	Suppression of 2-amino-1-methyl-6-phenylimidazo[4,5-*b*]pyridine-induced DNA damage in rat colon after grapefruit juice intake.	In vivo	Comet assay	[42]
2004	Influence of grapefruit juice intake on AFB_1_-induced liver DNA damage.	In vivo	Comet assay	[43]
2004	Capacity of the grapefruit juice to inhibit the rate of micronucleated polychromatic erythrocytes (MNPE) produced by daunorubicin.	In vivo	Micronucleus	[44]
2010	Capacity of the grapefruit juice to inhibit the rate of micronucleated polychromatic erythrocytes (MNPE) and Sister chromatid exchange (SCE) induced by ifosfamide.	In vivo	Micronucleus & SCE	[45]
2010	Capacity of 15 fruit juices to protect against the genotoxic effect produced by 2-amino-3-methylimidazo [4,5-*f*] quinoline and 2-amino-1-methyl-6-phenylimidazo [4,5-*b*] pyridine.	In vitro	Comet assay	[46]
2011	Capacity of grapefruit juice to inhibit the rate of micronucleated polychromatic erythrocytes (MNPE) in mice treated with benzo(a)pyrene.	In vivo	Micronucleus	[47]
2011	Evaluation of the genotoxic effect of H_2_O_2_ and the reduction of this damage by grapefruit juice in human lymphocytes.	In vitro	Comet assay	[48]
2013	Evaluation of the ameliorative role of grapefruit juice on the cytogenetic and testicular damage induced by the amiodarone in albino rats.	In vivo	Chromosomal aberrations	[49]
2014	Combination of rosemary and citrus bioflavonoids extracts from grape fruit against the genotoxicity induced by X-rays in human lymphocytes.	In vitro	Comet assay & Micronucleus	[50]
Phytochemical (naringin and naringenin)				
2001	Antigenotoxic effect of naringin against the damage induced by ifosfamide.	In vivo	Micronucleus	[51]
2003	Evaluation of the protective effect of naringin on H_2_O_2_-induced cytotoxicity and apoptosis in mouse leukemia P388 cells.	In vitro	Comet assay	[52]
2004	Effect of naringin on the cytotoxicity and apoptosis in mouse leukemia P388 cells treated with Ara-C.	In vitro	Comet assay	[53]
2007	Evaluation of the protective effect of naringin on the genomic damage induced by bleomycin.	In vitro	Micronucleus	[54]
2012	Influence of naringin on cadmium-induced genomic damage in human lymphocytes.	In vitro	Cromosomal aberrations & SCE	[55]

**Table 5:** Antigenotoxic studies of mangiferin (MGN).

Year	Main Objetive	Assay Employed	Reference
In vitro studies			
2014	Effect of MGN on gamma radiation-induced DNA damage in human lymphocytes and lymphoblastoid cells.	Comet assay	[74]
2012	Evaluation of protective effects of MGN against several mutagens (bleomycin, CP, AFB_1_, B[a]P, 2-AAF, H_2_O_2_, sodium azide, cisplatin, MMC, and DMNA).	Ames test, SOS Chromotest assay & Comet assay	[75]
2009	Cytoprotective and antigenotoxic potential of mangiferine against cadmium chloride CdCl_2_-induced toxicity in HepG2 cells.	Comet assay & Micronucleus	[76]
2011	Evaluation of the protector effect of MGN against methylmercury (MeHg) induced neurotoxicity by employing IMR-32 (human neuroblastoma) cell line.	Comet assay & Micronucleus	[77]
2011	Antigenotoxic potential of MGN against mercuric chloride (HgCl_2_)-induced genotoxicity in HepG_2_ cell line.	Comet assay & Micronucleus	[78]
2015	Evaluation of the Mangiferin to reduce etoposide-induced DNA damage in human umbilical cord mononuclear blood cells.	Comet assay & Micronucleus	[79]
In vivo studies			
2008	Protective role of mangiferin against B[a]P induced lung carcinogenesis in experimental animals.	Comet assay	[80]
2005	Ability of the mangiferin to reduce the frequency of radiation-induced micronucleated binucleate cells (MNBNCs) in cultured human peripheral blood lymphocytes.	Micronucleus	[81]
2010	The protective role of mangiferin (MGN) against cadmium chloride CdCl_2_-induced genotoxicity studied in Swiss albino mice.	Micronucleus	[82]

**Table 6:** Main research developed with berry fruits.

Year	Aim of the Study	Technique Used	Conclusion	Reference
In vitro studies				
2002	Protective effect of blueberries and blackberries against genotoxicity of 2-acetylaminofluorene (AAF) & 2-amino-1-methyl-6-phenylimidazo[4,5-*b*]pyridine (PhIP) in V79 cells.	CA	Genotoxic activity of AAF and PhIP was strongly reduced in a dose-related manner by berry fruits. Demonstrating that protection of fruits against genotoxicity of heterocyclic aromatic amines may take place within metabolically competent mammalian cells.	[86]
2010	Protective effect of the juices of blueberry, and blackberry against genotoxicity of 2-amino-3-methylimidazo[4,5-f]quinoline (IQ) &PhIP in V79 cells.	CA	These berry fruits showed a inhibitory effect on genotoxicity of both mutagens. The best protection was for the blueberry juice. As one possible mechanism of antigenotoxicity, is the inhibition of activating enzymes of xenobiotics, such as CYP1A2.	[46]
2013	Protective effect of blueberries against genotoxicity of *N*-methyl-*N*′-nitro-*N*-nitrosoguanidine (MNNG) & 7,12-dimethylbenz(a)anthracene (DMBA) assessed in HepG2.	MN	Blueberries were not toxic in vitro. The pre-treatment with blueberries reduced the micronucleus frequency induced by MNNG but the same effect is not present with DMBA.	[87]
2014	Protective effect of *Vaccinium myrtillus* extract against UVA- and UVB-induced damage in HaCaT cells.	MN & CA	The extract showed its free radical scavenging properties reducing oxidative stress and apoptotic markers, especially in UVA-irradiated cells.	[88]
In vivo studies				
2006	Protective effect of extract of *Vaccinium ashei* berries on DNA damage in the hippocampus and cerebral cortex, as well as on cognitive performance in mice.	CA	The extract reduced oxidative DNA damage in brain tissue. Suggesting that supplementation with V. ashei berries to mice improves the memory and has a protective effect on DNA damage, possibly due to the antioxidant activity.	[89]
2010	Protective effect of 8 weeks administration of a wild blueberry (*Vaccinium angustifolium*) against H2O2-induced DNA damage in lymphocytes of rats.	CA	The level of DNA damage was significantly lower in rats fed with the wild blueberry at the end of the eight weeks.	[90]
2012	Protective effect of cranberry ethanolic extract (CEE), (*Vaccinium macrocarpon*) against the DNA damage induced by B[a]P.	MN	The CEE was not genotoxic. On the contrary, reduces the frequency of micronucleus induced by B[a]P. Suggesting that the anticlastogenic effect of the CEE is related to the antioxidant capacity of the combination of phytochemicals present in its chemical composition.	[83]
2013	Protective effect of blueberries against genotoxicity of MNNG and DMBA assessed in polychromatic erythrocytes (PCEs) from the bone marrow of mice.	MN	Blueberries fruits reduced the frequency of micronucleus induced by MNNG and DMBA. However, in the case of DMBA, a dramatic reduction in the percentage of PCEs was observed, suggesting increased cytotoxicity.	[87]
Clinical Studies				
2007	Impact of multiple genetic polymorphisms on effects of a 4-week blueberry/apple juice intervention on ex vivo induced lymphocytic DNA damage in 168 healthy human volunteers.	CA	The analysis of 34 genetic polymorphisms showed that DNA damage is reduced faster in some individuals per share of micronutrients in the blueberry/apple juice.	[91]
2012	Protective effect blueberry (*Vaccinium corymbosum* L) on markers of oxidative stress and DNA damage induced by H_2_O_2_ in healthy volunteers.	CA	Blueberries significantly reduced H_2_O_2_-induced DNA damage in blood mononuclear cells. No significant differences were observed in peripheral arterial function and nitric oxide levels after blueberry intake. In conclusion, one portion of blueberries seems sufficient to improve cell antioxidant defense against DNA damage.	[92]
2015	Compare DNA damage in fresh versus cryopreserved peripheral blood mononuclear cells (PBMCs) obtained from subjects following a 6-week intervention with wild blueberry drink.	CA	The decrease in H_2_O_2_-induced DNA damage observed in the cryopreserved cells masked the protective effect of the wild blueberry drink documented in the fresh samples. Suggesting that H_2_O_2_-induced DNA damage could be significantly modified following the long-term storage of samples obtained from individuals participating in adietary intervention.	[93]

Comet Assay (CA), Micronucleus (MN).

**Table 7:** Antigenotoxic studies of β–glucan evaluated with the comet assay.

Year	Type of β-glucan	Type Cell	Mutagen or Carcinog	Reference
In vitro studies				
2003	β-glucan from *S. cerevisiae* β-glucan-chitin from *A. niger*	V79 hamster lung cells	H_2_O_2_	[110]
2006	Carboxymethyl chitin-glucan (CM-CG) from *A. niger*	Primary rat hepatocytes	BaP, *N*-nitrosomorpholine (NMOR) & dimethyldibenzocarbazole	[111]
2006	β-glucan from *A. brasiliensis*	Human peripheral lymphocytes	3-amino-1-methyl-5H-pyrido[4,3-b]indole, (+/−)-anti-B[a]P-7,8-dihydrodiol-9,10-epoxide & H_2_O_2_	[112]
2008	CM-CG from *A. niger*	Human hepatoma cells (HepG2) & V79 hamster lung cells	NMOR	[113]
2008	CM-CGfrom *A. niger*	Primary rat hepatocytes	H_2_O_2_ & 2,3-dimethoxy-1,4-naphthoquinone	[114]
2009	β-glucan from barley	HepG2	BaP	[115]
2009	β-glucan from *Agaricus blazei*	HepG2	BaP	[116]
2010	CM-CG from *A. niger*	HepG2 & HeLa cells	H_2_O_2_, Methylmethane sulfonate (MMS) & *N*-methyl-*N*′-nitro-*N*-nitrosoquanidine (MNNG)	[117]
2014	β-glucan from barley	HepG2	Radiation (6 Gy)	[118]
2014	β-glucan from *Ganoderma lucidum*	Human peripheral lymphocytes	Radiation (1, 2 & 4 Gy)	[119]
In vivo studies				
2004	CM-CG from *A. niger*	Lymphocytes, testicular cells & epithelial II cells from Sprague Dawley rats	H_2_O_2_	[120]
2011	CM-G from *S. cerevisiae*	Cells from patients with advanced prostate cancer (PCa)	Prostate cancer	[121]
2015	β-glucan from *S. cerevisiae*	Broiler chicken lymphocytes	AFB_1_	[122]
2015	β-glucan from *S. cerevisiae*	Mouse hepatocytes	AFB_1_	[102]
